# Upregulation of TLR2/4 Expression in Mononuclear Cells in Postoperative Systemic Inflammatory Response Syndrome after Liver Transplantation

**DOI:** 10.1155/2010/519589

**Published:** 2010-06-16

**Authors:** Ziqing Hei, Xinjin Chi, Nan Cheng, Gangjian Luo, Shangrong Li

**Affiliations:** Department of Anesthesiology, Third Affiliated Hospital, Sun Yat-sen University, Guangzhou 510630, China

## Abstract

*Background*. To explore the relationship between Toll-like rpheral blood mononuclear cells (PBMC) and systemic inflammatory response syndrome (SIRS) in postoperative patients of liver transplantation (LT). *Methods*. Blood samples of 27 patients receiving LT were collected at T1 (after induction of anaesthesia), T2 (25 minutes after the beginning of anhepatic phase), T3 (3 hours after graft reperfusion), and T4 (24 hours after graft reperfusion). The expression of TLR2/4 on PBMC and serum concentration of tumor necrosis factor (TNF)-*α*, interleukin (IL)-1*β*, and IL-8 were measured. The patients were divided into SIRS group (*n* = 12) and non-SIRS group (*n* = 15) for analysis. *Results*. Blood loss and transfusion were higher in the SIRS group than in the non-SIRS group. Both the preanhepatic and anhepatic phase were significantly longer in the SIRS group. The TLR2/4 expression on PBMC as well as serum TNF-*α*, IL-1*β*, and IL-8 were significantly higher at T3 and T4 than that at T1 and T2 in the SIRS patients. The expression of TLR4 on PBMC is positively correlated to serum TNF-*α*, IL-8. Expression of TLR2/4 on PBMC and serum concentrations of TNF-*α*, IL-1*β*, did not differ among the 4-time points in non-SIRS patients. *Conclusions*. Upregulation of TLR2/4 expression on PBMC may contribute to the development of postoperative SIRS during perioperative period of LT.

## 1. Introduction

Orthotopic liver transplantation (OLT) is the most effective and the best therapeutic solution for final stage liver diseases. More than 60,000 patients receive OLT every year worldwide [1]. Systemic inflammatory response syndrome(SIRS) often accompanies sepsis, trauma, hypoxia, and ischemia-reperfusion injury (IRI) [2–4]. The activation of mononuclear phagocytes, and consequently release of massive amount of proinflammatory cytokines may lead to multiorgan dysfunction syndrome (MODS) [3, 4]. SIRS is a common feature after major surgery [5, 6]. In recent years, SIRS has been interpreted as a warning sign for postoperative complications and organ failure [7–9]. Specifically, longer SIRS duration has been associated with poor outcomes after surgery [5, 6, 8, 9].

Toll-like receptors (TLRs) play an important role in many pathophysiological processes such as inflammation and IRI [10–14]. TLR2 and TLR4 are members of the TLRs family [13, 14], and could initiate inflammatory responses to various stimuli [2, 15]. 

A previous study in this laboratory revealed increased expression of TLR2/4 in mononuclear and proinflammatory cytokines in liver transplantation [16]. Based on this finding, we speculate that TLR2/4 may also contribute to the development of SIRS during OLT. In the current study, we examined the expression of TLR2/4 on PBMC in a group of OLT patients with SIRS, and compared the results to that in a group of patients without SIRS.

## 2. Patients and Methods

This study was approved by the Research Ethics Board of The Third Affiliated Hospital, Sun Yat-sen University. Written informed consent was obtained from all patients prior to the enrollment.

### 2.1. Study Population

Twenty-seven patients (24 males and 3 females) with end-stage liver diseases undergoing modified piggyback liver transplantation were enrolled. Among these patients, 12 had hepatitis B cirrhosis, 8 had small liver cancer (tumor diameter <3 centimeter) on a hepatitis B background, 4 had chronic severe hepatitis B and the remaining 3 had drug-related acute liver failure. Liver function based on modified Child-Pugh classification [17] was A in 10 patients, B in 5 patients, and C in the remaining 12 patients. Physical status of the patients was III or IV according to the American Society of Anesthesiologists (ASA) classification (Table 1).

The status of organ donors was cardiac death in 8 cases, brain death in 10 cases, and living relatives in 9 cases. Warm ischemia for donation after cardiac death was 3 to 4.5 minutes (Table 2). There was no warm ischemia in other cases.

### 2.2. Anesthesia

Anesthesia was induced with intravenous (i.v.) fentanyl and propofol. Tracheal intubation was facilitated with rocuronium. The lungs were mechanically ventilated with oxygen (50%). Partial pressure of carbon dioxide (P_ET_CO_2_) was maintained at 30–35 mmHg. Anesthesia was maintained with isoflurane and intermittent i.v. of fentanyl. Blood pressure was maintained with dopamine infusion if necessary. Body temperature was keep at 36~37°C by a convective air warming system and fluid warming system. To avoid interference of measurements of TLR2/4 expression on mononuclear cells, no whole blood was transfused during operation. Only concentrated red blood cells, fresh frozen plasma and cryoprecipitate were transfused during operation according to monitoring.

### 2.3. Surgical Procedure

All patients received modified piggyback liver transplantation with venous reformation and no veno-venous bypass (VVBP). Surgical management of first and second hepatic hilums is similar to classic orthotopic liver transplantation but without short hepatic vein disposal. After disconnection of the first hepatic hilum, vena cava was interrupted by a satinskys clamp from the back of the liver. Vena cava of second hepatic hilum was blocked by a Klintmalm liver clamp. The liver was then removed. The openings of hepatic veins on the anterior wall of the vena cava were connected to form an open inverted triangular cuff. The posterior wall of the donor inferior vena cave (IVC) was incised to fashion a wide-open inverted triangular cuff that matched the IVC opening in the recipient. The openings were closed with 4–0 Prolene suture. The graft was flushed with 400 to 800 ml cold FFP. Donor infrahepatic vena cava was ligated, and the portal vein was anastomosized. The clamps were then removed to allow reperfusion. Hepatic artery and bile duct anastomoses were completed [18–20]. 

The entire procedure consisted of an anhepatic phase (from vascular clamping to reperfusion of portal vein and inferior vena cava) and a neohepatic phase (from reperfusion of donor liver to the end of operation).

### 2.4. Collection of General Data

The demographic data as well as the Child-Turcotte-Pugh (CTP) scores, ASA classification, duration of the operation, volume of blood loss and input were collected. Duration of postoperative mechanical ventilation, alanine aminotransferase (ALT), aspartate aminotransferase (AST), prothrombin time (PT), blood urea nitrogen (BUN), and serum creatinine (SCr) were also recorded.

### 2.5. Collection of Blood Samples

Whole blood (4 mL) was collected at T1 (after induction of anaesthesia), T2 (25 minutes after the beginning of anhepatic phase), T3 (3 hours after graft reperfusion), and T4 (24 hours after graft reperfusion). Two mL of blood sample was collected in EDTA tubes for analysis of TLR2/4 immediately with flow cytometry. The remaining two mL of blood sample was collected in dry tubes for TNF-*α*, IL-1*β*, and IL-8 assay.

### 2.6. Analysis of TLR2/4 Expression

Twenty *μ*L allophycocyanin (APC) antihuman CD14 (eBioscience) plus 20 *μ*L fluoresceinisothiocyanate (FITC) antihuman Toll-like receptor 2 (eBioscience, San Diego, California, USA) or phycoerythrin (PE) antihuman Toll-like receptor 4 (eBioscience) were added to 100 *μ*L of EDTA treated blood. The mixture was incubated for 20 minutes in the dark at ambient temperature. After which, they were mixed with 2 mL of RBC Lysis Buffer (eBioscience) in the dark for 15 minutes and then centrifuged for 5 minutes at 300 g. After rinsing twice, the supernatant was discarded and sample was preserved at 4°C in the dark. Samples were quantified with FACs Calibur flow cytometry (Becton Disckinson, Franklin Lakes, New Jersey, USA). The isotype controls were FITC mouse IgG2*α* and PE mouse IgG2*α* (eBioscience). 

### 2.7. Cytokine Assay

TNF-*α*, IL-1*β*, and IL-8 were measured with ELISA (Rapidbio, West Hills, California, USA).

### 2.8. Perioperative SIRS Monitoring

Patients' temperature, heart rate, respiratory rate, and white blood cell count were assessed every 6 hours for 7 days after the operation. The diagnosis of SIRS was based on the presence of two or more of the following criteria [21], verified by an ICU physician as well as an anesthesiologist: (1) temperature >38°C or <36°C, (2) heart rate >90 per minute, (3) respiratory rate >20 per minute or PaCO_2_ < 32 mmHg, and (4) white blood cell count >12,000/mL, <4,000/mL, or >10% immature (band) forms.

### 2.9. Data Analysis

The data are expressed as mean ± standard deviation. One-Way ANOVA was used to analyze the difference between the different phases in the same group. Independent-samples *t*-test was used to analyze the difference between the SIRS and non-SIRS groups. Data of nonnormal distribution are expressed as median (interquartile range) [Median (Q)], and were analyzed by Wilcoxon signed ranks test. Spearman correlation analysis was used to determine the relationship between different measures. *P* < .05 is considered statistically significant. All data were processed by SPSS12.0 for windows (SPSS Inc., Chicago, Ill, USA).

## 3. Results

### 3.1. General Data

Twelve out of 27 patients developed SIRS after OLT (at 6 to 78 hours), and two died of lung infection. There were no significant differences between two groups on graft origination, duration of cold or warm ischemia (Table 2). Blood loss and concentrated red blood cell (RBC) transfusion during the operation were larger in the SIRS group than in the non-SIRS group. The pre-anhepatic phase and anhepatic phase lasted longer in SIRS patients (Table 3). CTP score, age, gender, body weight, ascites, urinary production, the length of neohepatic phase or the entire operation did not differ between the SIRS and non-SIRS groups (Table 3). 

Duration of postoperative mechanical ventilation in the SIRS group was significantly longer than non-SIRS group. Hepatic function, renal function, and infection (respiratory tract) after the surgery also did not differ between the 2 groups (Table 4).

### 3.2. Difference of TLR2/4 Expression on PBMC between SIRS and Non-SIRS Groups

**Figure 1:**
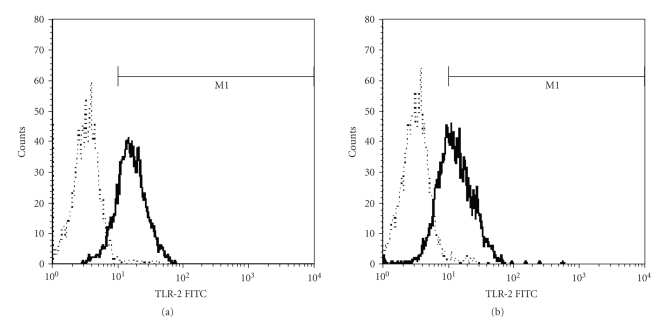
Representative FACS plots of TLR2 staining on PBMC. (a) SIRS; (b) non-SIRS. The non-specific binding is relatively small relative to specific binding as defined by the isotype controls. Dotted line represents the isotype control.

**Figure 2:**
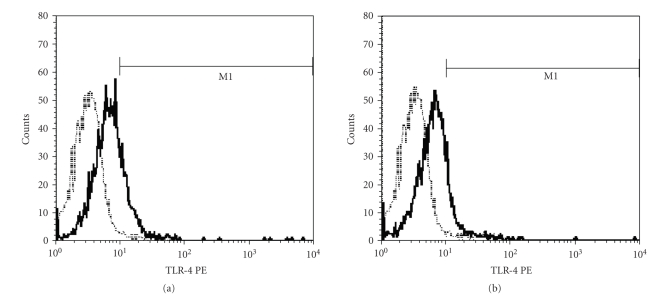
Representative FACS plots of TLR4 staining on PBMC. (a) SIRS; (b) non-SIRS. The non-specific binding is relatively small relative to specific binding as defined by the isotype controls. Dotted line represents the isotype control.

**Figure 3:**
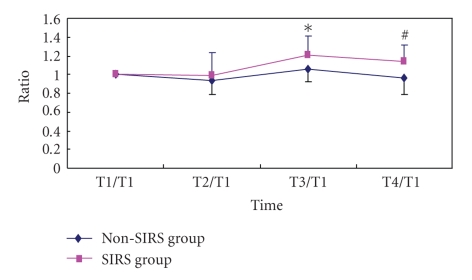
Upregulation of TLR2 expression on PBMC. Mean ± SD (*n* = 12 for SIRS; *n* = 15 for non-SIRS); *P < .01,  ^#^ *P* < .05, compared with T1/T1.

**Figure 4:**
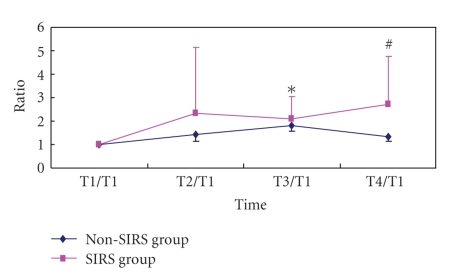
Upregulation of TLR4 expression on PBMC. Mean ± SD (*n* = 12 for SIRS; *n* = 15 for non-SIRS); *P < .01,  ^#^ *P* < .05, compared with T1/T1.

The baseline TLR2 on PBMC was 74% (interquartile range: 25%) and 80% (interquartile range: 28%) in SIRS and non-SIRS groups (*P* > .05, Figure 1). Baseline TLR4 was 12% (interquartile range: 8%) and 18% (interquartile range: 21%) in SIRS and non-SIRS groups (*P* > .05, Figure 2). TLR 2 expression was significantly higher at T3 and T4 in comparison to T1 and T2 in the SIRS patients but not in the non-SIRS group (Figure 3). Similar changes were found for TLR4 expression (Figure 4).

The expression of TLR2/4, and particularly relative increase at T3/4 over T1, in two died patients were higher than the average.

### 3.3. Difference of the Serum Levels of TNF-*α*, IL-1*β* and IL-8 between SIRS and Non-SIRS Groups

**Figure 5:**
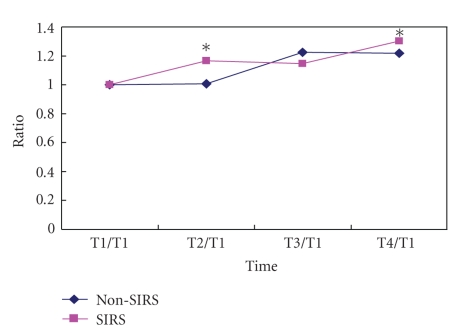
Increase of serum TNF-*α*. Median (Q) *n* = 12 for SIRS; (*n* = 15 for non-SIRS); *P < .05, compared with T1/T1.

**Figure 6:**
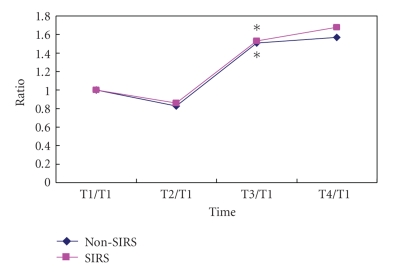
Increase of serum IL-1*β*. Median (Q) (*n* = 12 for SIRS; *n* = 15 for non-SIRS); *P < .05, compared with T1/T1.

**Figure 7:**
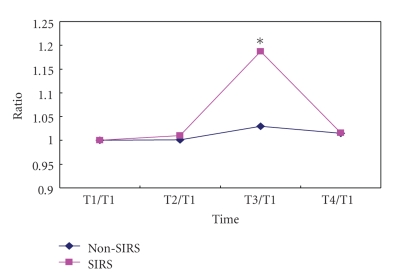
Increase of serum IL-8. Median (Q) (in SIRS group, *n* = 12; in non-SIRS group, *n* = 15); *P < .05, compared with T1/T1.

Baseline TNF-*α*, IL-1*β*, and IL-8 was 90 (interquartile range: 118), 34(interquartile range: 239), and 163(interquartile range: 181) pg/mL in SIRS group, and 96(interquartile range: 488), 38(interquartile range: 161), and 64(interquartile range: 173) pg/mL in the non-SIRS group, respectively. Serum TNF-*α*, IL-1*β*, and IL-8 was significantly higher at T3 and T4 in comparison to T1 and T2 in the SIRS group, but not in the non-SIRS group (Figures 5, 6, and 7).

### 3.4. Correlation Analysis

There was no relationship between TLR2/4 and CTP score. The expression of TLR4, but not TLR2, was positively correlated to serum TNF-*α* (*r* = 0.310,  *P* = .029), and IL-8 (*r* = 0.304,  *P* = .025) in the SIRS group but not in the non-SIRS group. In the SIRS patients, the increase of TLR4 at T4 was positively correlated with the length of anhepatic phase (*r* = 0.688,  *P* = .013).

## 4. Discussion

The current study demonstrated that expression of TLR2/4 on PBMC and concentration of inflammatory cytokines after liver transplant reperfusion were significantly higher in patients with SIRS than those without. 

SIRS is an inflammatory state caused by serious trauma, and infection [2–4, 21]. A cardinal feature of SIRS is the activation of inflammatory cells such as monocyte-macrophages, neutrophils, and massive release of proinflammatory cytokines [2, 4]. SIRS is common in OLT due to surgical trauma, hemorrhage, and ischemia-reperfusion injury. Incidence of postoperative SIRS in our study is 44%. 

TLR2/4 on immune cells can activate nuclear factor kappa B (NF-*κ*B) and activator protein-1 (AP-1) in response to a variety of pathological conditions, which in turn initiate or amplify inflammation, and ultimately, organ injury [11–14]. Lipopolysaccharide (LPS) can induce TLR4 gene expression in granulocyte and endothelial cells, and activate NF-*κ*B and the production of TNF-*α*, IL-6, and IL-8 [22]. TLR4 antibody can inhibit activation of NF-*κ*B and production of inflammatory cytokines [23]. Previous studies also demonstrated that high expression of TLR4 is positively correlated with ischemia-reperfusion injury [24]. Importantly, the transcriptional and translational signal of TLR2/4 in mononuclear cell was upregulated significantly in SIRS patients [25]. 

Although there was no significant difference in CTP scores between the SIRS and non-SIRS groups, the expression of TLR2/4 on PBMC and serum proinflammatory cytokines at T3 and T4 were significantly higher in the SIRS group. The increase of TNF-*α*, IL-1*β* or IL-8 was also positively correlated with the expression of TLR4 in monocytes in the SIRS group, suggesting that high expression of TLR2/4 in OLT patients is associated with SIRS. 

Previous studies indicated that cytokine, endotoxin are involved in the regulation of TLR2/4 expression [26–30]. Within the context of liver transplantation, factors that could upregulate TLR2/4 may include prolonged surgery, massive blood loss and transfusion, liver ischemia/reperfusion, translocation of enteric microbes during portal vein occlusion and reopening, and ischemia-reperfusion injury of graft [31–33]. Specifically, blood loss, the length of preanhepatic phase and anhepatic phase may be the most important factors for upregulation of TLR2/4 expression in PBMC after OLT. 

In conclusion, our findings suggest that upregulation of TLR2/4 on PBMC could initiate SIRS after major surgery such as OLT.

## Figures and Tables

**Table 1:** Basic characteristics of the study population.

	Total patient (*n*)	27
	Gender (M : F)	24 : 3
	Age (years)	47 ± 11
	Weight (kilogram)	64 ± 10
	CTP score (A : B : C)	10 : 5 : 12
Diagnosis	hepatitis B virus cirrhosis (*n*)	12
	hepatitis B virus cirrhosis accompanied with small liver cancer (*n*)	8
	chronic severe hepatitis B (*n*)	4
	acute liver failure induced by drug (*n*)	3
	Physical Status (III : IV)	10 : 17

Mean ± SD or median (Q).

**Table 2:** Postoperative SIRS in patients receiving different categories of transplant.

	SIRS			non-SIRS		
	*n*	Warm ischemia times (min )*	Cold ischemia times^# (h)	*n*	Warm ischemia times (min)	Cold ischemia times (h)
No heart beat donors	3	4.3 ± 0.6	5.0 ± 2.6	5	4.4 ± 0.4	6.4 ± 0.5
Living-related donors	5	0	1.0 ± 0.3	4	0	1.3 ± 0.5
Brain dead donors	4	0	2.0 ± 0.8	6	0	1.3 ± 0.5

Total	12	0 (3)	1.8 (2)	15	0 (4)	1.5 (5)

Mean±SD or median (Q).

*Warm ischemia time was defined as the time between discontinuation of blood perfusion and initiation of aortic or hepatic arterial perfusion with the cold preservation solution.

^#^Cold ischemia time was defined as the time from aortic or hepatic arterial perfusion with cold preservation solution until reperfusion.

**Table 3:** Clinical characteristics in SIRS versus non-SIRS during OLT.

Features	Total	SIRS	non-SIRS
*n*	27	12	15
Gender (M : F)	24 : 3	11 : 1	13 : 2
Age (years)	47 ± 11	48 ± 11	47 ± 12
Weight (kilogram)	64 ± 10	61 ± 8	66 ± 11
CTP score (A : B : C)	10 : 5 : 12	4 : 2 : 6	6 : 3 : 6
Ascites (mL)	622 ± 1305	775 ± 983	500 ± 1538
Urine (mL)	1541 ± 699	1455 ± 567	1609 ± 801
Volumes of blood loss (mL)	3011 ± 1286	3617 ± 1380*	2527 ± 1004
Concentrated red blood cell (mL)	989 ± 536	1225 ± 554*	800 ± 453
Pre-anhepatic phase (min)	105 ± 44	125 ± 55*	89 ± 24
Anhepatic phase (min)	46 ± 22	55 ± 30*	38 ± 8
Neohepatic phase (min)	252 ± 46	242 ± 26	259 ± 57
Total operation time (min)	402 ± 65	422 ± 65	386 ± 63

Mean ± SD or median (Q), *P < .05, compared with non-SIRS.

**Table 4:** Postoperative clinical characteristics in SIRS versus non-SIRS.

	SIRS	non-SIRS
	(*n* = 12)	(*n* = 15)
Duration of mechanical ventilation (h)	19 (44)*	11 (9)
ALT (U/L)	530 ± 268	655 ± 410
AST (U/L)	136 (153)	60 (112)
PT (s)	17 ± 4	16 ± 2
BUN (mmol/L)	19 ± 11	13 ± 6
Cr (*μ*mol/L)	102 ± 56	92 ± 40
Pulmonary infecttion	9/12	6/15

Mean ± SD or median (Q), *P < .05, compared with the non-SIRS group.

ALT = alanine aminotransferase; AST = aspartate aminotransferase; PT = prothrombin time; BUN = blood urea nitrogen; SCr = serum creatinine.

Duration of postoperative mechanical ventilation in the SIRS group was significantly longer than non-SIRS group. Hepatic function, renal function and pulmonary infecttion after the surgery also did not differ between the 2 groups.
